# Ultrasound for Preoperatively Predicting Pathology Grade, Complete Cytoreduction Possibility, and Survival Outcomes of Pseudomyxoma Peritonei

**DOI:** 10.3389/fonc.2021.690178

**Published:** 2021-09-15

**Authors:** Lei Liang, Xuedi Han, Nan Zhou, Hongbin Xu, Jun Guo, Qian Zhang

**Affiliations:** ^1^Department of Ultrasound, Aerospace Center Hospital, Beijing, China; ^2^Department of Myxoma, Aerospace Center Hospital, Beijing, China; ^3^Department of Gastroenterology, Beijing Friendship Hospital, Beijing, China

**Keywords:** pseudomyxoma peritonei, ultrasound, sonography, prognosis, recurrence

## Abstract

**Objectives:**

This study aimed to investigate the value of using ultrasound (US) preoperatively for predicting pathological classification, complete cytoreduction possibility, and survival rate of patients with pseudomyxoma peritonei (PMP).

**Methods:**

We retrospectively studied PMP patients who were scheduled for cytoreductive surgery between May 2009 and October 2019. US examination was performed before surgery. Factors related to high-grade pathology and poor completeness of cytoreduction (CC) score were identified. Associations between ultrasound characteristics and the survival status were also examined to identify independent predictive factors.

**Results:**

PMP patients with clear ascites, abdominal lymph nodes, omental cake, abdominal mass, portal infiltration, and mesenteric involvement visible on US were considered to have high-grade pathology. Various US features were shown to be independent prognostic markers for inadequate cytoreduction in PMP patients. Portal infiltration and mesenteric involvement were significant prognostic factors for lower survival rates (hazard ratio = 3.092, 3.932, respectively). A visual nomogram including these factors was constructed to predict survival rates. The consistency index was 0.777, which reflected relatively high accuracy.

**Conclusions:**

Preoperative US has the potential to predict pathological grade and resectability of PMP. Portal infiltration and mesenteric involvement were independent predictors of poor clinical outcomes in PMP patients. Furthermore, a simple-to-use nomogram derived from our study data may be a helpful visual tool in clinical practice to predict 1-, 2-, and 3-year survival rates for PMP patients.

## Introduction

Pseudomyxoma peritonei (PMP) is a condition that is characterized by grossly disseminated intraperitoneal mucinous epithelial neoplasms and gelatinous ascites composed of copious amounts of mucin (1–3). Histopathological, immunohistochemical, and molecular-genetic data have suggested that the vast majority of PMP cases develop from mucinous carcinomas in the appendix (4, 5).

The recommended treatment for PMP is cytoreductive surgery (CRS) plus hyperthermic intraperitoneal chemotherapy (HIPEC) (6). This combination treatment is undoubtedly the optimal strategy associated with improved clinical outcomes. CRS aims to decrease the tumor burden in the abdominal cavity as much as possible, which is the key to treatment efficacy (7). HIPEC is delivered following CRS to clear any microscopic lesions. It is recommended that the completeness of the cytoreduction (CC) score is assessed immediately at the end of the CRS. Due to the high rate of surgical morbidity, candidate patients should be selected carefully. Lee et al. (8) reported that determining the extent of the clinical pathological classification and CC score is crucial to assessing survival outcomes. These findings should be considered when explaining to patients before surgery the benefits and risks of CRS. Thus, preoperative imaging assessment of the pathological grade and CC score is of great clinical significance.

### Objective

Most PMP patients are recommended to undergo ultrasound (US) examination because of the nonspecific signs of abdominal distension and abdominal pain. The value of US is in its quantitative evaluation of PMP. This study aimed to identify preoperative ultrasonic predictors of pathological grade, resectability, and survival of PMP patients. Additionally, we sought to develop tools to determine survival time. To address this, we developed and validated a nomogram to predict mortality.

## Materials and Methods

### Study Population

This was a prospective study of 502 PMP patients with a confirmed histological diagnosis of PMP. All patients were aged 18 years or over and underwent CRS between May 2009 and October 2019 at the Aerospace Center Hospital. US examination was conducted 2 weeks before surgery. As an optimal preoperative staging investigation, preoperative computed tomography (CT) scan was routinely performed in all patients. The study was approved by the ethics committee of the Aerospace Center Hospital. All the enrolled patients signed the informed consent. All enrolled patients were followed until February 1, 2020.

### Inclusion and Exclusion Criteria

The inclusion criteria were as follows: 1) underwent CRS from May 2009 to October 2019; 2) diagnosed with PMP based on surgery or pathology; and 3) US images could be obtained in the preoperative examination 2 weeks prior to surgery at the Aerospace Center Hospital. Patients who were lost to follow-up were excluded.

### Procedure and Follow-Up

Patients were hospitalized with a high suspicion of PMP based on imaging findings. Initially, PMP was judged and classified by ascitic fluid cytology examination. This tool is rarely applied for diagnostic purposes; however, there are currently no cytological diagnostic guidelines. Lu et al. (9) reported that mucinous epithelial and mucinous deposits in pathology cell block and smears were effective in diagnosing and classifying PMP. In cases where ascites were thick and could not be extracted, core needle biopsy of the mass and omental cake was used for diagnosis instead. The final diagnosis and classification were confirmed by postoperative pathological findings in accordance with the fourth edition of the World Health Organization (WHO) classification system (10, 11). The classification of low- and high-grade diseases was based on the morphologic characteristics like architecture, cytology, presence of signet ring cells, and mitotic activity (12).

Patients were scheduled for CRS at the Aerospace Center Hospital, and the quality of CRS was defined according to the Sugarbaker’s CC score based on tumor distribution. No tumor or a residual tumor of less than 2.5 mm was scored as CC-0 (complete cytoreduction) or CC-1 (optimal cytoreduction), respectively. A residual tumor larger than 2.5 mm was scored as CC-2/3 and was considered as inadequate cytoreduction. Tolerance should be considered in the decision to undergo HIPEC treatment decision. However, only a very small number of patients refused HIPEC treatment due to personal reasons.

Six months after discharge, patients underwent blood tests and biannual imaging reexaminations. Routine follow-up involved an annual CT scan and serum tumor biomarker measurements.

### US Examination

For US diagnosis, we used ultrasonic diagnostic instruments (C6-1 convex array probe, frequency 1–6 MHz; L10-2 linear array probe, frequency 2–10 MHz) (Supersonic Imagine, France; SAMSUNG, South Korea; Philips, Netherlands). The entire abdomen was scanned using C6-1 and L10-2 probes. The location, size, extent, border, and echogenicity were recorded if ascites, omental cake, liver scalloping, celiac, pelvic masses, or enlarged lymph nodes were present. Ascites were classified into three subtypes according to appearance: clear ascites, which were relatively anechoic, and no septum could be found in the area by US; mucus granules ascites, which showed multiple punctate high-level echoes in echo-free ascites that moved under the pressure of the transducer; and jelly-like ascites, which were gelatinous ascites with a positive fluctuation test. To assess the presence of particulates or the mobility of ascites, posture adjustment and the probe pressing manner were applied. The adjacent viscera were also scanned to exclude organ invasion. The sonographic images were reviewed by two doctors with expertise in PMP imaging with extensive 10 years of experience, who were blinded to patient data.

### Statistical Analysis

Continuous variables were described as median and interquartile range (IQR). Categorical variables were described as frequencies and percentages. Univariate and multivariate logistic regression models were used to identify the factors related to high-grade pathology and high CC score. The receiver operating characteristic (ROC) curves, sensitivities, specificities, and 95% confidence intervals (CIs) were calculated to evaluate the diagnostic performance using MedCalc statistical software (MedCalc Software Ltd., Belgium). The survival curves were plotted using a Kaplan–Meier method in GraphPad Prism (GraphPad Software, USA), and a log-rank test was used for statistical analysis. The associations between US characteristics and the survival status were examined using univariate and multivariate backward Cox proportional hazard regression models. All statistical tests were two-sided, and a significance level of 0.05 was used. Data analyses were performed using SAS software (SAS Institute Inc., USA).

A multivariate Cox regression model was used for the development of the nomogram. A simple-to-use nomogram provided a convenient graphical representation of the prediction model of survival probability that is useful for patient evaluation. Each independent variable was scored according to its contribution to the outcome. The scores of these variables were summed to provide a prediction of survival rates.

## Results

### The Demographic, Clinical, and US Characteristics of Enrolled Patients

**Figure 1:**
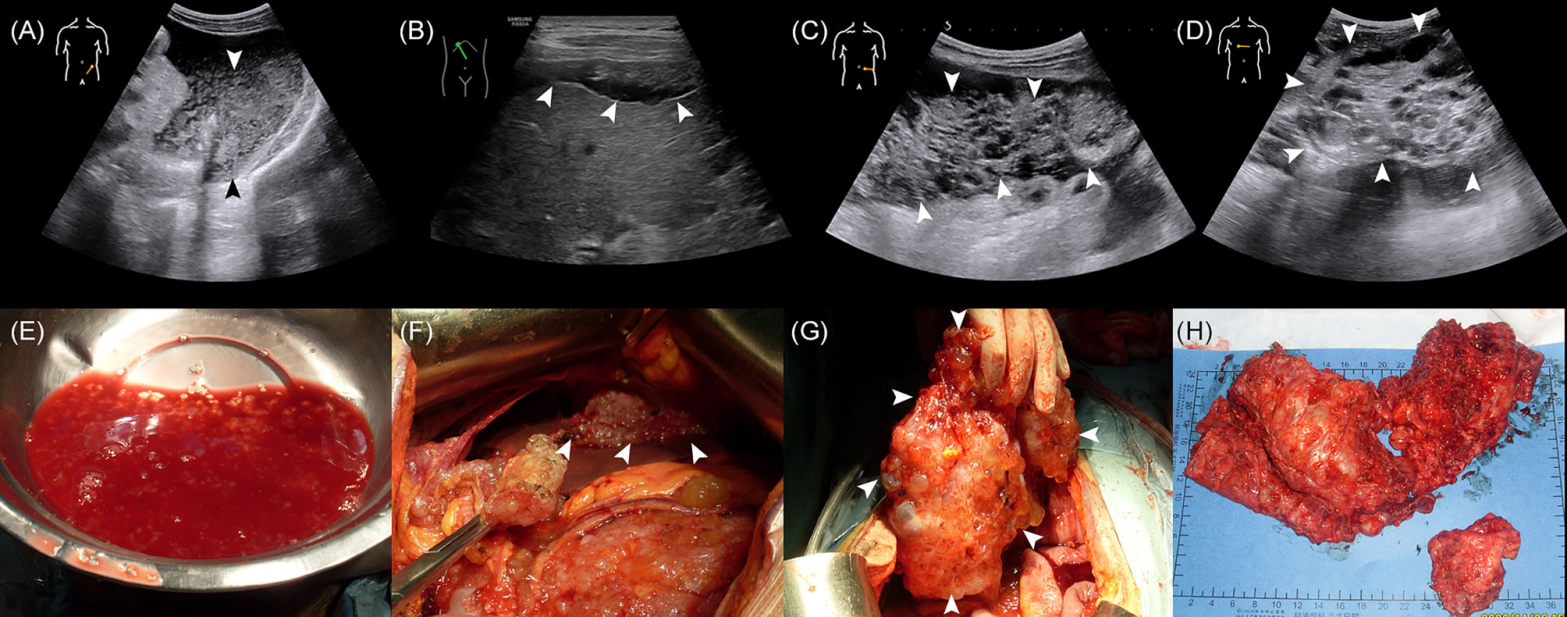
The US characteristics and the corresponding gross specimens of PMP patients. **(A, E)** The absence of mucus granules ascites. **(B, F)** Liver scalloping. **(C, G)** Omental cake. **(D, H)** Abdominal mass.

**Figure 2:**
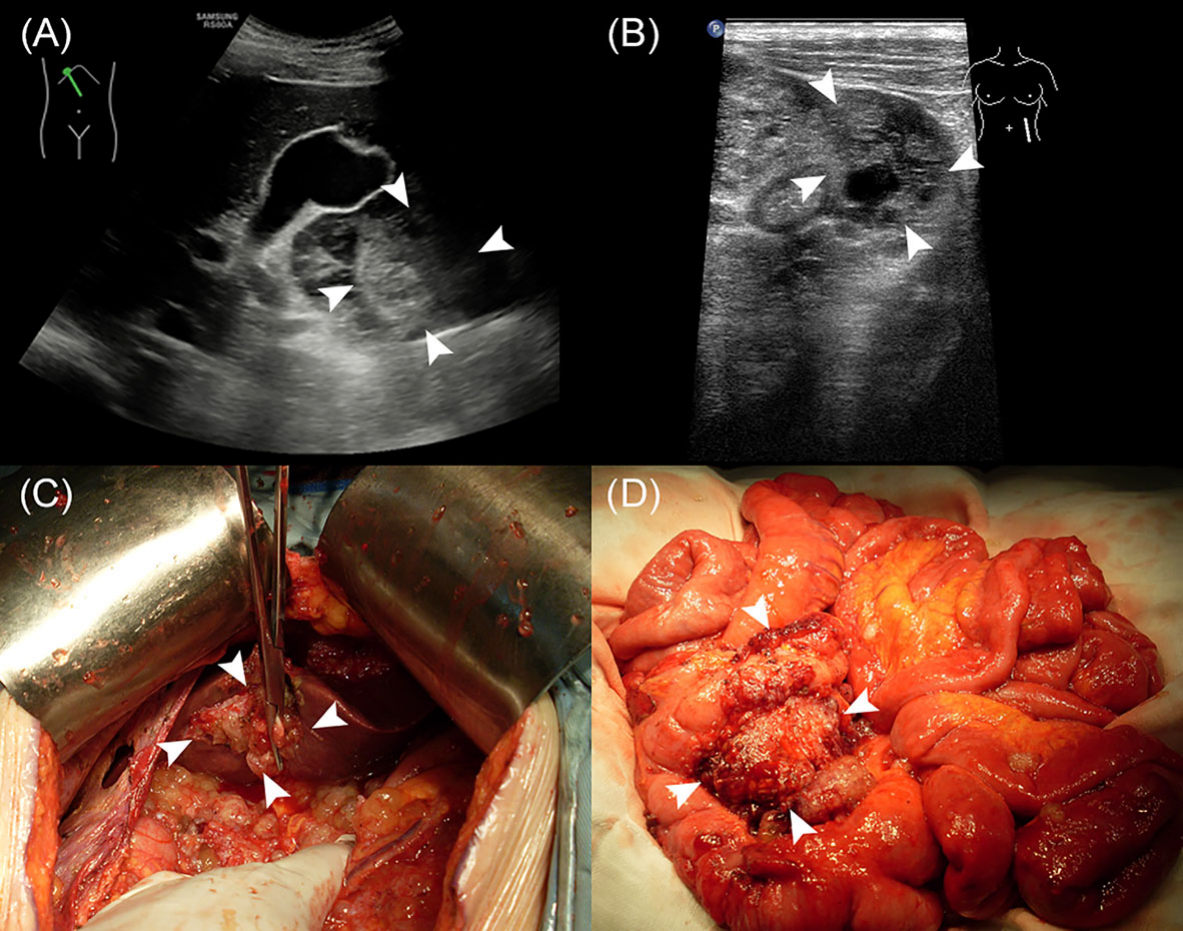
The US characteristics and the corresponding gross specimens of PMP patients. **(A, C)** Portal infiltration. **(B, D)** Mesenteric involvement.

The median age of the 502 enrolled patients was 58 years with an IQR of 49 to 64 years. The majority of patients (63.15%) were female. Among the PMP patients, 35.06% had no ascites under US examination, whereas 22.51% had clear ascites, 14.54% had mucus granule ascites (**Figures 1A, E**), and 27.89% had jelly-like ascites. Liver scalloping was observed in 73.90% of patients (**Figures 1B, F**), 47.01% of patients showed significant omental cake (**Figures 1C, G**), 41.63% showed the abdominal mass (**Figures 1D, H**), and 61.75% showed involvement of lesser omental bursa. Most patients had no abdominal lymph nodes (98.21%) (**Table 1**). Portal infiltration (**Figures 2A, C**) occurred in 58.37% of patients, and mesenteric involvement (**Figures 2B, D**) was observed in 17.33% of patients. These characteristics of PMP could also be visualized on CT scans. The detection ability of preoperative US and preoperative CT for PMP was compared, as **Table 2** depicts. Compared with CT, US had higher accuracy in displaying ascites (*p* < 0.001), liver scalloping (*p* < 0.001), omental cake (*p* < 0.001), and lesions in lesser omental bursa (*p* < 0.001) but lower accuracy than CT for identifying portal infiltration and mesenteric involvement. Furthermore, US had an advantage in distinguishing mucus granules ascites from mucus over CT (*p* < 0.001).

**Table 1:** Demographic, clinical, and US characteristics of patients.

Characteristic	Median (IQR)/N (%) total = 502
Age, years	58 (49–64)
Gender	
Male	185 (36.85)
Female	317 (63.15)
Astices	
No	176 (35.06)
Clear	113 (22.51)
Mucus granules	73 (14.54)
Jelly-like	140 (27.89)
Liver scalloping	
No	131 (26.10)
Yes	371 (73.90)
Abdominal lymph nodes	
No	493 (98.21)
Yes	9 (1.79)
Omental cake	
No	266 (52.99)
Yes	236 (47.01)
Abdominal mass	
No	293 (58.37)
Yes	209 (41.63)
Portal infiltration	
No	209 (41.63)
Yes	293 (58.37)
Mesenteric involvement	
No	415 (82.67)
Yes	87 (17.33)
Lesions in lesser omental bursa	
No	192 (38.25)
Yes	310 (61.75)
Intraoperative PCI	
PCI < 10	104 (20.72)
10 ≤ PCI ≤ 20	68 (13.55)
PCI > 20	330 (65.74)
CC score	
CC-0/1	251 (50.00)
CC-2/3	251 (50.00)
HIPEC	
Yes	436 (86.85)
No	66 (13.15)
Origin	
Appendix	479 (95.42)
Others	23 (4.58)
Pathology	
Low-grade	349 (69.52)
High-grade	153 (30.48)

**Table 2:** The diagnostic accuracy of US and CT with surgery as the gold standard.

	CT	US	p
Ascites	82.67%	96.02%	<0.001
Ascites granules	57.57%	98.80%	<0.001
Liver scalloping	89.44%	96.22%	<0.001
Abdominal lymph nodes	85.06%	76.89%	0.003
Omental cake	69.92%	89.04%	<0.001
Abdominal mass	57.57%	93.23%	<0.001
Portal infiltration	80.88%	69.92%	<0.001
Mesenteric involvement	86.65%	57.37%	<0.001
Lesions in lesser omental bursa	65.14%	85.26%	<0.001

### US Characteristics Related to Pathological Grade and CC Score

Age and gender showed no significant relation to pathological grade. The US characteristics that were significantly associated with pathological grade were ascites (*p* < 0.001), abdominal lymph nodes (*p* = 0.022), omental cake (*p* < 0.001), abdominal mass (*p* = 0.007), portal infiltration (*p* = 0.008), and mesenteric involvement (*p* < 0.001) (**Table 3**). Moreover, we identified a strong correlation between CC score and presence of clear ascites (*p* = 0.004), liver scalloping (*p* = 0.005), abdominal mass (*p* = 0.022), portal infiltration (*p* < 0.001), mesenteric involvement (*p* < 0.001), and lesions in the lesser omental bursa (*p* = 0.003) (**Table 4**).

**Table 3:** The univariate and multivariate logistic regression for pathology.

Logistic regression for pathology	Univariate regression		Multivariate regression	
	Odds ratio (95% CI)	*p* value	Odds ratio (95% CI)	*p* value
Age, years	1.002 (0.986–1.019)	0.775	–	–
Gender	
Female	1			
Male	0.908 (0.611–1.349)	0.632	–	–
Ascites	
No	0.418 (0.256–0.685)	0.001	0.339 (0.187–0.613)	<0.001
Clear	1		1	
Mucus granules	0.273 (0.139–0.537)	<0.001	0.310 (0.151–0.640)	0.002
Jelly-like	0.325 (0.190–0.556)	<0.001	0.309 (0.172–0.553)	<0.001
Liver scalloping	
No	1			
Yes	0.749 (0.490–1.144)	0.181	–	–
Abdominal lymph nodes	
No	1		1	
Yes	19.20 (2.380–154.892)	0.006	11.873 (1.433–98.369)	0.022
Omental cake	
No	1		1	
Yes	0.564 (0.382–0.832)	0.004	0.261 (0.153–0.444)	<0.001
Abdominal mass	
No	1		1	
Yes	1.485 (1.012–2.179)	0.043	1.798 (1.172–2.759)	0.007
Portal infiltration	
No	1		1	
Yes	1.588 (1.069–2.358)	0.022	2.026 (1.198–3.427)	0.008
Mesenteric involvement	
No	1		1	
Yes	2.872 (1.788–4.612)	<0.001	2.742 (1.567–4.799)	<0.001
Lesions in lesser omental bursa	
No	1			
Yes	1.413 (0.948–2.107)	0.090	–	–

**Figure 3:**
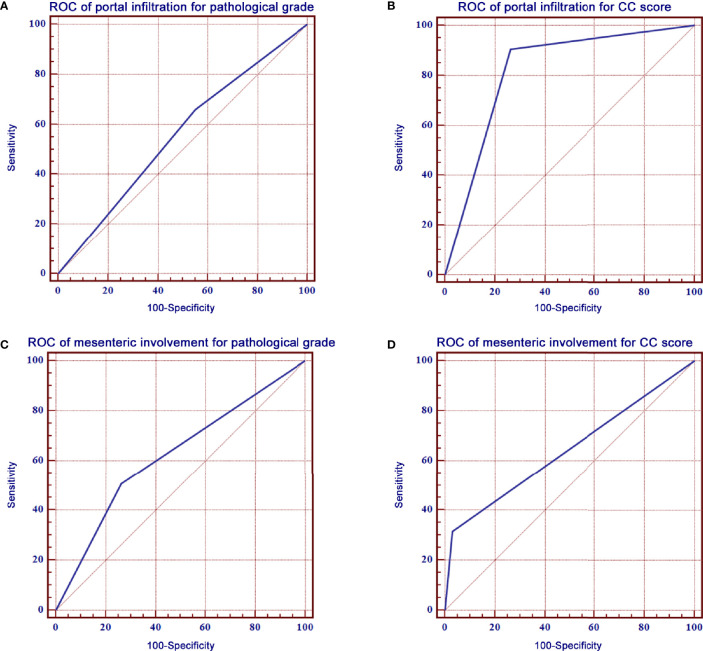
ROC curves of portal infiltration, mesenteric involvement for pathological grade, and CC score. **(A)** ROC curve of portal infiltration showed a sensitivity of 66.0% and a specificity of 45.0% for pathological grade. **(B)** ROC curve of portal infiltration had a sensitivity of 90.4% and a specificity of 73.7% for the CC score. **(C)** ROC curve of mesenteric involvement showed a sensitivity of 66.0% and a specificity of 45.0% for pathological grade. **(D)** ROC curve of mesenteric involvement had a sensitivity of 90.4% and a specificity of 73.7% for CC score.

The ROC curve of portal infiltration in relation to pathological grade showed a sensitivity of 66.0% (95% CI: 57.9%–73.5%) and a specificity of 45.0% (95% CI: 39.7%–50.4%) (**Figure 3A**). The ROC curve of portal infiltration in relation to the CC score showed a sensitivity of 90.4% (95% CI: 86.1%–93.8%) and a specificity of 73.7% (95% CI: 67.8%–79.0%) (**Figure 3B**). Mesenteric involvement was difficult to detect in some cases but represented high specificity in predicting the CC score and survival outcomes once detected by the US. The ROC curve of mesenteric involvement in relation to pathological grade showed a sensitivity of 28.8% (95% CI: 21.7%–36.6%) and a specificity of 87.7% (95% CI: 83.8%–90.9%) (**Figure 3C**). Mesenteric involvement in relation to CC score showed a sensitivity of 31.5% (95% CI: 25.8%–37.6%) and a specificity of 96.8% (95% CI: 93.8%–98.6%) (**Figure 3D**).

### US-Documented Portal Infiltration and Mesenteric Involvement as Survival Risk Factors

**Figure 4:**
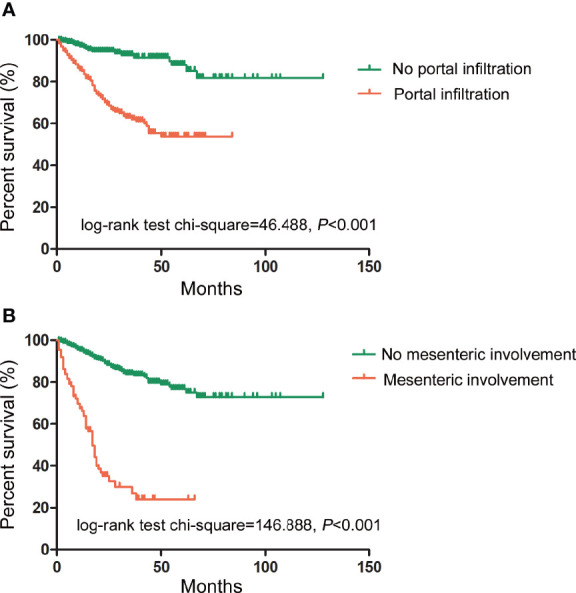
Prognostic impact of portal infiltration and mesenteric involvement as detected by US on survival outcomes. **(A)** The log-rank test of survival showed that patients with portal infiltration had lower survival duration (in months) than patients with no portal infiltration. (*χ* ^2^ = 46.488, *p* < 0.001). **(B)** The log-rank test of survival showed that patients with mesenteric involvement also had lower survival duration than those without mesenteric involvement. (*χ* ^2^ = 146.888, *p* < 0.001).

The relationship between US characteristics and the outcomes of PMP patients was evaluated in the multivariate Cox proportional hazard regression model. In addition, the pathological grade, the resectability of treatment (CC score), intraoperative peritoneal cancer index (PCI), and tumor origin were investigated. In contrast to portal infiltration (*p* **** < 0.001) and mesenteric involvement (*p* < 0.001), several other US characteristics showed no significant relationship with the survival rates, as shown in **Table 4**. The log-rank test of survival rate showed that patients with portal infiltration or mesenteric involvement had poorer survival rates (χ**^2^** = 46.488, 146.888, *p* **** < 0.001) than patients with no portal infiltration or mesenteric involvement (**Figure 4**). The results indicated that portal infiltration or mesenteric involvement was associated with poor prognosis (hazard ratio [HR] = 3.092, [95% CI: 1.795–5.328], HR = 3.932, [95% CI: 2.646–5.844]). The absence of HIPEC (*p* **** < 0.001) and high-grade pathology (*p* **** < 0.001) were both independent predictors of poor survival rates. Patients who did not receive HIPEC and those with high-grade pathology had a shorter duration of survival, with an HR of 2.677 (95% CI: 1.794–3.993) and 2.529 (95% CI: (1.758–3.636), respectively. There were no significant correlation between the intraoperative PCI, CC score, tumor origin, and survival outcomes in the multivariate analysis (*p* **** > 0.05).

**Table 4:** The univariate and multivariate logistic regression for CC score.

Logistic regression for CC score	Univariate regression		Multivariate regression	
	Odds ratio (95% CI)	*p* value	Odds ratio (95% CI)	*p* value
Age, years	1.013 (0.998–1.029)	0.091	–	–
Gender	
Female	1			
Male	1.648 (1.143–2.377)	0.008	–	–
Ascites	
No	0.113 (0.065–0.195)	<0.001	0.337 (0.161–0.707)	0.004
Clear	1		1	
Mucus granules	1.805 (0.916–3.556)	0.088	1.945 (0.821–4.604)	0.130
Jelly like	0.832 (0.495–1.397)	0.486	1.184 (0.601–2.330)	0.625
Liver scalloping	
No	1		1	
Yes	7.989 (4.828–13.220)	<0.001	2.947 (1.377–6.309)	0.005
Abdominal lymph nodes	
No	1			
Yes	0.797 (0.212–3.005)	0.738	–	–
Omental cake	
No	1			
Yes	6.889 (4.651–10.202)	<0.001	–	–
Abdominal mass	
No	1		1	
Yes	0.685 (0.480–0.979)	0.038	0.541 (0.320–0.916)	0.022
Portal infiltration	
No	1		1	
Yes	26.512 (15.985–43.970)	<0.001	8.806 (4.782–16.216)	<0.001
Mesenteric involvement	
No	1		1	
Yes	13.951 (6.571–29.622)	<0.001	7.570 (2.932–19.543)	<0.001
Lesions in lesser omental bursa	
No	1		1	
Yes	15.917 (9.881–25.642)	<0.001	2.624 (1.380–4.988)	0.003

**Figure 5:**
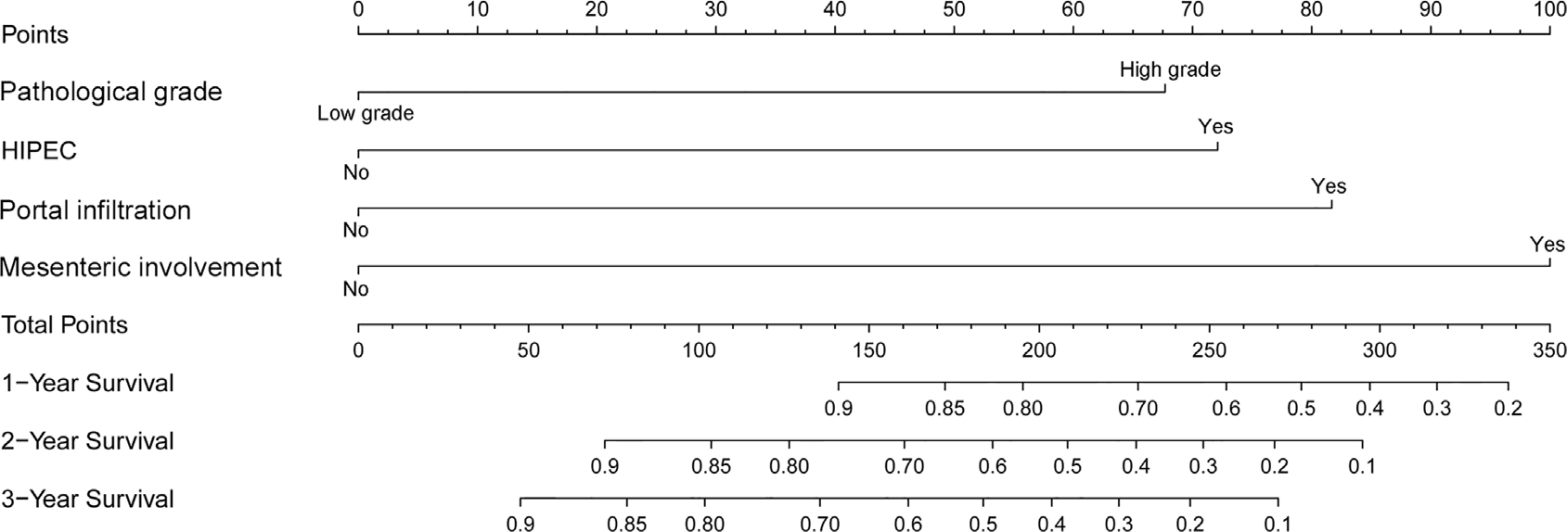
A nomogram to predict the 1-, 2-, and 3-year survival rates in PMP patients. It is used by drawing a line perpendicular from the corresponding axis of each risk factor until it reaches the top line labeled “Points.” Then, the number of points for all risk factors is summed, and a line is drawn that descends from the axis labeled “Total points” until it intercepts each of the survival axes to determine the 1-, 2-, and 3-year survival probabilities.

**Figure 6:**
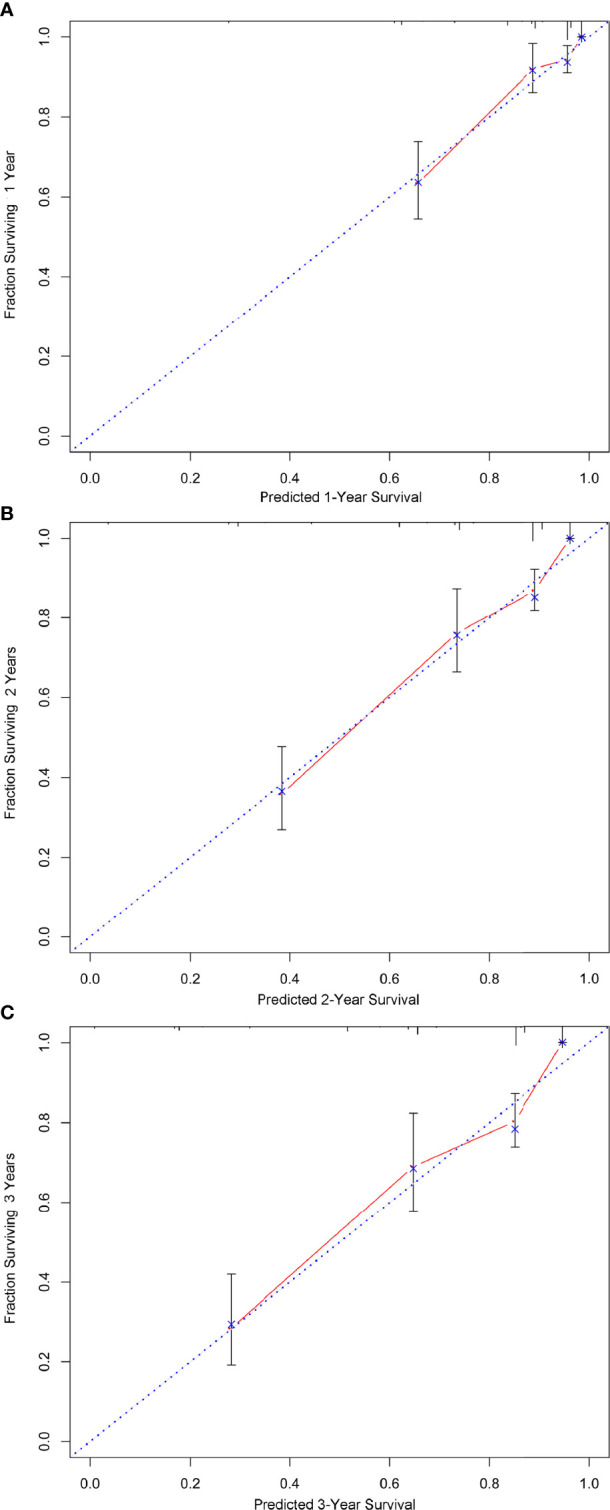
The calibration of the nomograms for predicting 1-, 2-, and 3-year survival probabilities. **(A–C)** show the nomograms of 1-, 2-, and 3-year survival probabilities, respectively. Plots show that the predicted survival rates closely corresponded with the actual survival rates, which are represented by the dotted lines.

A nomogram was developed using a multivariate Cox regression model to predict the 1-year, 2-year, and 3-year survival rates, helping predict the survival probability in a visual format (**Figure 5**). Pathological grade, HIPEC, portal infiltration, and mesenteric involvement were included in the nomogram because the Cox regression model predicted these factors as independent covariates for the risk of death. A patient**’**s probability of individual survival was calculated by adding the scores of these selected variables. The consistency index for the nomogram was 0.777, which reflected a relatively high accuracy in predicting the survival probability. Furthermore, the similarities between the nomogram**’**s predicted survival rates and the actual survival rates were evaluated in 1-, 2-, and 3-year validation plots. The bootstrapped resample (500 iterations) was used to verify the accuracy of the nomogram. These plots showed that the predicted survival rates closely corresponded with the actual survival rates represented by the dotted lines (**Figure 6**).

## Discussion

### The Utility of Preoperative US

PMP develops from mucinous carcinomas that mostly arise from the appendix and/or ovary. According to the appearance of the ascites, the proportion of mucinous substances can be classified as clear, mucinous particles or jelly-like ascites. As a common imaging method, CT is routinely used for the preoperative evaluation of PMP patients (13). However, the small peritoneal tumors in the ascites or small septa in a mass may be indistinguishable, resulting in a decrease in diagnostic. Furthermore, the extent of disease assessed by CT consistently underestimates peritoneal spread in PMP patients due to the partial volume effect and the limited resolution of soft tissue. US is the first imaging modality in most hospitals for investigating unclear abdominal problems and is recommended for PMP patients with primary symptoms of abdominal distension and pain. As an imaging technique, US is superior to CT because of its low cost, availability, and tolerability. However, the use of US in PMP patients has not been sufficiently investigated.

Several studies (14–16) have confirmed the feasibility and advantages of using US in the quantitative evaluation of PMP. Our findings showed that US provides an excellent visualization of the PMP lesions. The diagnostic accuracy of US and CT was compared to the gold standard of surgery (**Table 5**). The fundamental physics underlying US lies in the difference in acoustic impedance within tissues. This offers significant advantages in observing the distribution of the lesions and allows the boundary between the ascites and the organs to be observed clearly. Furthermore, the boundary of the mucinous abdominal mass can be clearly detected. Predicting the distribution of disease accurately before surgery is valuable and is beneficial for performing a successful operation. However, due to the increased abdominal girth of PMP patients, deep exploration depth, and the imaging of intestinal gas of PMP patients, detecting mesenteric involvement by US was shown to have low sensitivity. Nevertheless, high specificity could be achieved once mesenteric involvement was detected by US.

**Table 5:** The Cox regression model with US characteristics.

Cox regression model for death	Univariate regression		Multivariate regression	
	Hazard ratio (95% CI)	*p* value	Hazard ratio (95% CI)	*p* value
Age, years	1.008 (0.993–1.025)	0.295	–	–
Gender	
Female	1			
Male	1.138 (0.792–1.635)	0.483	–	–
Ascites	
No	0.402 (0.245–0.661)	<0.001		
Clear	1		–	–
Mucus granules	0.854 (0.513–1.420)	0.543	–	–
Jelly like	0.597 (0.374–0.953)	0.031	–	–
Liver scalloping	
No	1			
Yes	1.696 (1.059–2.715)	0.028	–	–
Abdominal lymph nodes	
No	1			
Yes	1.407 (0.447–4.429)	0.559	–	–
Omental cake	
No	1			
Yes	1.548 (1.079–2.221)	0.018	–	–
Abdominal mass	
No	1			
Yes	0.987 (0.686–1.419)	0.005	–	–
Portal infilitration	
No	1		1	
Yes	5.036 (3.005–8.440)	<0.001	3.092 (1.795–5.328)	<0.001
Mesenteric involvement	
No				
Yes	7.238 (5.000–10.479)	<0.001	3.932 (2.646–5.844)	<0.001
Lesions in lesser omental bursa	
No	1			
Yes	2.654 (1.693–4.159)	<0.001	–	–
Intraoperative PCI	1.057 (1.036–1.079)	<0.001	–	–
CC score	
CC–0/1	1			
CC–2/3	2.585 (1.744–3.831)	<0.001	–	–
HIPEC	
Yes	1		1	
No	3.976 (2.696–5.863)	<0.001	2.677 (1.794–3.993)	<0.001
Origin	
Appendix	1			
Others	1.686 (0.823–3.454)	0.154	–	–
Pathology	
Low-grade	1		1	
High-grade	3.086 (2.157–4.416)	<0.001	2.529 (1.758–3.636)	<0.001

### Diagnostic Value of US in Pathology

Our results suggested that preoperative US contributed to the prediction of the pathological grade of PMP. Compared with clear ascites, mucinous particles in ascites and jelly-like ascites were more suggestive of a low-grade subgroup. A recent study (17) also showed that the mucus content of the high-grade subgroup was lower than that of the low-grade group, which was attributed to pathological and cytological differences. In the low-grade subgroup, the appendiceal neoplasm epithelial cells were positioned in a single row and the mucin secretion was typically exuberant, whereas the cells in the high-grade group were belt-shaped, island, or cribriform-shaped with severe dysplasia. The secretory function of the columnar cells was severely damaged, which led to a decrease in mucus secretion (2, 6, 18). Moreover, the concentrated tumor mass resulted in liver scalloping and omental cake (19, 20). The current study results also demonstrated that the presence of omental cake predicted a higher pathological grade.

A previous investigation revealed that enlarged lymph nodes can sometimes exist in peritoneal mucinous carcinomas (21). The lesions were extensively distributed throughout the abdominal cavity in PMP patients; however, hematogenous or lymphatic metastasis rarely occurred (22, 23). Although rare, we detected lesions with lymph node involvement that occurred in nine high-grade cases in this study. Lymph node metastasis was correlated with high-grade pathology according to the univariate and multivariate analyses.

### Diagnostic Value of US in Assessing the Feasibility of Complete Cytoreduction

Recent studies (24, 25) have indicated that preoperative imaging techniques, such as CT and magnetic resonance imaging (MRI), could predict the resectability of PMP. Additionally, some US manifestations were significant prognostic factors for the resectability in PMP cases. The univariate analysis showed that omental cake was a poor prognostic factor for CC score. However, clear ascites, the appearance of liver scalloping, and abdominal masses were independent predictors of a higher CC score, which indicated a greater likelihood of incomplete cytoreduction. US showed lesions in the lesser omental bursa in the cases of gastric involvement, predicting a poorer CC score. Preoperative US evaluations of resectability helped to optimize the selection of patients who should undergo CRS, which could prevent additional unnecessary surgeries. These findings should be considered when explaining benefits and risks to patients before surgery.

### Predictive Value of US in Survival Rates

The present study indicated that porta hepatis and the mesenterium were involved in the high-grade group. However, the multivariate analysis showed that only these two US features were highly correlated with the resectability of the lesions as well as the survival rates. This finding was comparable with that of a previous literature (26). The porta hepatis had complex anatomical relationships with the hepatic artery, portal vein, common bile duct, gallbladder triangle, and other important anatomical structures. It was difficult to clear all the hepatoduodenal and hepatogastric ligament tumors which severely affected the outcome of surgery. It demonstrated that the presence of residual unresectable disease in porta hepatis may influence the prognosis (25) and might explain why the involvement of the porta hepatis predicted PMP outcomes. The evaluation of porta hepatis using two-dimensional US has previously been reported to be associated with the unresectability of PMP; however, to the best of our knowledge, no study has reported its predictive role for CC score and survival rates. The present study indicated that portal infiltration helped in the early determination of resectability and survival rates.

Multivariate analysis identified mesenteric involvement as an independent predictor of a poorer CC score and survival rates. It was impossible to achieve extensive surgical resection for optimal treatment owing to irresectable small-bowel involvement, which was consistent with the study reported by Mittal et al. (2). In the present study, high-grade pathology and not undergoing HIPEC were also identified as independent predictors of poorer survival rates according to the multivariate analysis, which was consistent with previous reports (27). A simple-to-use nomogram that included these independent factors was developed using our study data, which might be a useful visualization tool for predicting 1-, 2-, and 3-year survival probabilities for PMP in clinical practice.

When variables were examined using a univariate analysis, the CC score was associated with the survival rates. Maximal cytoreduction offered a greater chance of long-term survival. However, the multivariate analysis showed that the CC score was not a significant independent factor for survival outcomes. This was in contrast to the study reported by Chua et al. (28) and Ma et al. (29), which demonstrated that CC score was independently correlated with overall survival. The differences in patient factors and previous surgical or medical therapies between our results and their reports may contribute to the discrepant findings.

### Limitations

This research had several limitations. First, although the PMP patient population was recruited from the core hospital for PMP surgery, our study was still a single-center study in a relatively small cohort. Secondly, although all US procedures and evaluations were performed by two doctors with 10 years of PMP examination experience, US is highly dependent on an individual skill level. This may reduce the repeatability of US results compared with that of the CT results to some extent. Finally, we had no data on urological or vascular involvement. We aim to acquire additional data for further study in the future.

## Conclusion

Preoperative US has the potential value in predicting pathological grade, complete cytoreduction possibility, and the benefits of the surgery. Portal infiltration and mesenteric involvement are independent predictors of poorer survival rates. Moreover, a simple-to-use nomogram can effectively predict 1-, 2-, and 3-year survival of PMP patients, which may be considered for clinical utility.

## Data Availability Statement

The raw data supporting the conclusions of this article will be made available by the authors, without undue reservation.

## Ethics Statement

The studies involving human participants were reviewed and approved by the Ethics Committee of Aerospace Center Hospital. The patients/participants provided their written informed consent to participate in this study. Written informed consent was obtained from the individual(s) for the publication of any potentially identifiable images or data included in this article.

## Author Contributions

All authors contributed to the study conception and design. Material preparation was performed by XH, NZ, and HX. Data collection and analysis were performed by XH, NZ, and QZ. The first draft of the manuscript was written by XH and LL. QZ and JG revised the manuscript critically for important intellectual content, and all authors commented on previous versions of the manuscript. All authors contributed to the article and approved the submitted version.

## Funding

This work was supported by the Gold-Bridge Funds for Beijing (grant number ZZ21054), Aerospace Center Hospital Foundation (grant number YN201710), Beijing Talents Fund (grant number 2018000021469G198), and Natural Science Foundation of Beijing Municipal (grant number 7204249).

## Conflict of Interest

The authors declare that the research was conducted in the absence of any commercial or financial relationships that could be construed as a potential conflict of interest.

## Publisher’s Note

All claims expressed in this article are solely those of the authors and do not necessarily represent those of their affiliated organizations, or those of the publisher, the editors and the reviewers. Any product that may be evaluated in this article, or claim that may be made by its manufacturer, is not guaranteed or endorsed by the publisher.

## Abbreviations

PMP, pseudomyxoma peritonei; CRS, cytoreductive surgery; HIPEC, hyperthermic perioperative chemotherapy; CC, completeness of cytoreduction; US, ultrasound; CT, computed tomography; MRI, magnetic resonance imaging; HR, hazard ratio; PCI, peritoneal cancer index; ROC, receiver operating characteristic.
